# Skeletal muscle fiber hypercontraction induced by *Bothrops asper* myotoxic phospholipases A_2_ *ex vivo* does not involve a direct action on the contractile apparatus

**DOI:** 10.1007/s00424-023-02840-w

**Published:** 2023-07-20

**Authors:** Alfredo Jesús López-Dávila, Natalie Weber, Arnab Nayak, Leon Fritz, Kian Rami Moustafa, Luis Vincens Gand, Enke Wehry, Theresia Kraft, Thomas Thum, Julián Fernández, José María Gutiérrez, Bruno Lomonte

**Affiliations:** 1 Institute of Molecular and Cell Physiology, Hannover Medical School, Carl-Neuberg-Str. 1, 30625 Hannover, Germany; 2 Institute of Molecular and Translational Therapeutic Strategies, Hannover Medical School, Carl-Neuberg-Str. 1, 30625 Hannover, Germany; 3 Instituto Clodomiro Picado, Facultad de Microbiología, Universidad de Costa Rica, San José, 11501 Costa Rica

**Keywords:** Myotoxin, Snake venom, Phospholipase A_2_, Muscle, Hypercontraction

## Abstract

Myonecrosis is a frequent clinical manifestation of envenomings by Viperidae snakes, mainly caused by the toxic actions of secreted phospholipase A_2_ (sPLA_2_) enzymes and sPLA_2_-like homologs on skeletal muscle fibers. A hallmark of the necrotic process induced by these myotoxins is the rapid appearance of hypercontracted muscle fibers, attributed to the massive influx of Ca^2+^ resulting from cell membrane damage. However, the possibility of myotoxins having, in addition, a direct effect on the contractile machinery of skeletal muscle fibers when internalized has not been investigated. This question is here addressed by using an *ex vivo* model of single-skinned muscle fibers, which lack membranes but retain an intact contractile apparatus. Rabbit psoas skinned fibers were exposed to two types of myotoxins of *Bothrops asper* venom: Mt-I, a catalytically active Asp49 sPLA_2_ enzyme, and Mt-II, a Lys49 sPLA_2_-like protein devoid of phospholipolytic activity. Neither of these myotoxins affected the main parameters of force development in striated muscle sarcomeres of the skinned fibers. Moreover, no microscopical alterations were evidenced after their exposure to Mt-I or Mt-II. In contrast to the lack of effects on skinned muscle fibers, both myotoxins induced a strong hypercontraction in myotubes differentiated from murine C2C12 myoblasts, with drastic morphological alterations that reproduce those described in myonecrotic tissue *in vivo*. As neither Mt-I nor Mt-II showed direct effects upon the contractile apparatus of skinned fibers, it is concluded that the mechanism of hypercontraction triggered by both myotoxins in patients involves indirect effects, i.e., the large cytosolic Ca^2+^ increase after sarcolemma permeabilization.

**Supplementary Information:**

The online version contains supplementary material available at 10.1007/s00424-023-02840-w.

## Introduction

Envenomings by snakes of the family Viperidae are characterized, among other clinical manifestations, by prominent skeletal muscle necrosis [13, 41]. This effect is primarily due to the direct action of myotoxic secreted phospholipase A_2_ (sPLA_2_) enzymes and sPLA_2_ homologs on skeletal muscle fibers [16, 22]. These two types of viperid myotoxins, which belong to the group II sPLA_2_s [37], differ in their ability to hydrolyze phospholipids, owing to variations in residues at the catalytic site and the Ca^2+^-binding loop. Myotoxic Asp49 sPLA_2_s have the canonical catalytic residues of this group of secreted enzymes and are, therefore, enzymatically active [32]. In contrast, sPLA_2_ homologs present substitutions at position 49 (numbering according to [36]), where a Lys substitutes Asp in most cases, and in some residues comprising the Ca^2+^-binding loop [12, 27]. As a consequence, these variants are devoid of enzymatic activity [6, 11]. Despite this variation in their catalytic properties, both types of proteins induce skeletal muscle necrosis [17, 22].

Studies carried out *in vivo*, in cell culture models, and with artificial membranes have addressed the mechanism of action of these two variants of myotoxic sPLA_2_s. In both cases, there is a rapid disruption of the plasma membrane of muscle fibers *in vivo* and of myotubes in culture, as well as of liposomes, followed by a rapid series of intracellular degenerative events in the first two models [9, 14, 17, 23, 40]. In the case of Asp49 sPLA_2_s, myotoxicity depends on phospholipid hydrolysis, since inhibition of catalysis abolishes their action on muscle cells [33]. In the case of Lys49 sPLA_2_-like homologs, plasma membrane disruption occurs in the absence of phospholipid hydrolysis and is likely to depend on the ability of these toxins to bind and disrupt the integrity of membranes through the action of cationic and hydrophobic residues [10, 22]. Interestingly, these two types of myotoxins act synergistically [34], hence providing an adaptive advantage for enhanced activity on muscle tissue. In addition to the direct interaction and disruption that these toxins exert in the plasma membrane of muscle fibers, there is evidence indicating that they are also internalized [31, 38], although the implications of such internalization for the mechanisms of cell damage have not been elucidated.

The initial disruption in the integrity of the plasma membrane leads to a number of cellular derangements. The main functional consequence of such alteration is a prominent influx of Ca^2+^ [8, 40], which, in turn, unleashes a series of intracellular events such as myofilament hypercontraction and mitochondrial alterations [16, 17, 32]. However, it is unknown whether the hypercontraction of myofilaments is only due to the increase of cytosolic Ca^2+^ or whether the toxins, once internalized, are able to directly affect the contractile apparatus. An experimental strategy to address this question is based on the use of isolated skinned fibers which are devoid of membranes but maintain an intact contractile apparatus [18].

A previous study used this experimental platform to address the action of a myotoxic Lys49 sPLA_2_ homolog on rat cardiomyocytes [29]. It was observed that this myotoxin induced hypercontraction of cardiomyocytes associated with plasma membrane disruption and increases in cytosolic Ca^2+^ levels without directly affecting the contractile apparatus. Since the main target of myotoxic sPLA_2_s is skeletal muscle, in the present study we assessed the effect of a myotoxic Asp49 sPLA_2_ (Mt-I) and a Lys49 sPLA_2_ homolog (Mt-II) on skinned skeletal muscle fibers prepared from rabbit psoas muscle. In parallel, we assessed the action of these myotoxins on myotubes in culture. Our findings show that both myotoxins induce cytotoxicity associated with myofilament hypercontraction in myotubes in culture, but do not have a direct effect on the contractile apparatus in skinned muscle fibers.

## Material and methods

### Isolation of myotoxins

Myotoxin-I (Mt-I; Uniprot P20474) is an Asp49 sPLA_2_ enzyme, while Myotoxin-II (Mt-II; UniProt P24605) is a Lys49 sPLA_2_-like protein, both isolated from the venom of *Bothrops asper* of adult specimens collected in the Pacific versant of Costa Rica. Isolation of the toxins was performed by ion-exchange chromatography on CM-Sephadex C-25 [24] followed by reverse-phase HPLC on a semi-preparative C_8_ column, as described previously [33].

### Solutions for mechanical experiments

Solutions for mechanical experiments were prepared as previously described [4, 5]. All solutions were adjusted to pH 7.0. Free Ca^2+^ concentrations were expressed as pCa (−LOG 10[Ca^2+^]) and were in the range of 7.50 (relaxing solution) to 4.47 (activating solution). The skinning solution contained 5.0 mM KH_2_PO_4_, 3.0 mM Mg-acetate, 5.0 mM EGTA, 1.0 mM Na_2_ATP, 50 mM Na-creatine phosphate, 5.0 mM NaN_3_, 10 mM glutathione, 2.0 mM dithiothreitol, and a protease inhibitor cocktail. Pre-rigor solution (22°C) contained 10 mM imidazole, 2.5 mM EGTA, 7.5 mM EDTA, and 135 mM potassium propionate. Rigor solution (22°C) contained 10 mM imidazole, 2.5 mM EGTA, 2.5 mM EDTA, and 150 mM potassium propionate. Relaxing and activating solutions (5°C) contained 10 mM imidazole, 2.0 mM MgCl_2_, 1.0 mM MgATP, 1.0 mM EGTA (or CaEGTA), 50 mM sodium creatine phosphate, and 500 U/mL of creatine kinase. Solutions with different pCa values were obtained by mixing appropriate volumes of relaxing and activating solutions. For positive control experiments, increasing concentrations of the myosin inhibitors para-amino-blebbistatin (AmBleb; 6.2, 12.5, 25, and 50 μM) and 2,3-Butandione 2-monoxime (BDM; 10, 20, 30, 40 and 50 mM) were added to activating solution. Chemicals were obtained from Sigma-Aldrich Chemie GmbH (Munich, Germany). AmBleb was obtained from Motorpharma Ltd. (Budapest, Hungary).

### Preparation of single-skinned muscle fibers for mechanical experiments

Single fast-twitch muscle fibers were prepared as previously described [2, 43]. Briefly, fiber bundles were isolated from rabbit psoas muscles and incubated for 30 min at 4°C in a skinning solution containing 0.5 % Triton X-100. The skinned fiber bundles were thereafter equilibrated in Triton X-100 free skinning solution containing 3 M sucrose and a protease inhibitor cocktail. The skinned bundles were subsequently shock-frozen in liquid propane and stored in liquid nitrogen in screw cap tubes. For experiments, skinned fiber bundles were thawed in a sucrose-free skinning solution containing a fresh protease inhibitor cocktail. Single-skinned muscle fibers were thereafter gently isolated with forceps and kept for up to 4 days at 4°C without a detectable loss of function [20, 21].

### Protocol for mechanical experiments

The methodology and the apparatus used to perform mechanical experiments on isometric-held single-skinned fibers have been previously described [2, 3]. Briefly, single-skinned fibers (8–10 mm long) were mounted between the force transducer and a length driver of the apparatus using cyanoacrylate glue (Histoacryl; B. Braun Surgical GmbH, Melsungen, Germany). The ends of the fibers were thereafter stiffened with glutaraldehyde, using pre-rigor and rigor solutions as previously described [20]. The sarcomere length was set to 2.4 μm in a relaxing solution by means of laser diffraction [2, 3]. The mounted fiber was transferred to activating solution (saturating Ca^2+^ concentration, pCa 4.47) and maximal isometric force (Force_max_) and the rate constant of isometric force redevelopment after a short period of isotonic shortening (*K*_TRmax_) were measured. To this end, the fiber was cycled every 5 s between isometric steady-state contraction and short (330 ms) periods of unloaded isotonic shortening with subsequent restretch to the initial (isometric) sarcomere length; see [28, 30]. Subsequently, the isometric force was also measured at different pCa values corresponding to several sub-saturating Ca^2+^ concentrations. *K*_TRmax_ values were obtained by fitting force transients obtained at pCa 4.47 using a single-exponential function. Force−pCa relations were fitted using a sigmoidal Hill equation:

$${F}_n(pCa)=\frac{1}{1+{10}^{n_H\left({pCa}_{50}- pCa\right)}}$$

where Fn is the force at pCa = −LOG10 [Ca^2+^] normalized to maximum force at pCa 4.47, pCa_50_ is pCa when Fn = 0.5, and *n*_H_ is the steepness (Hill coefficient) of the force−pCa relation.

**Fig. 1:**
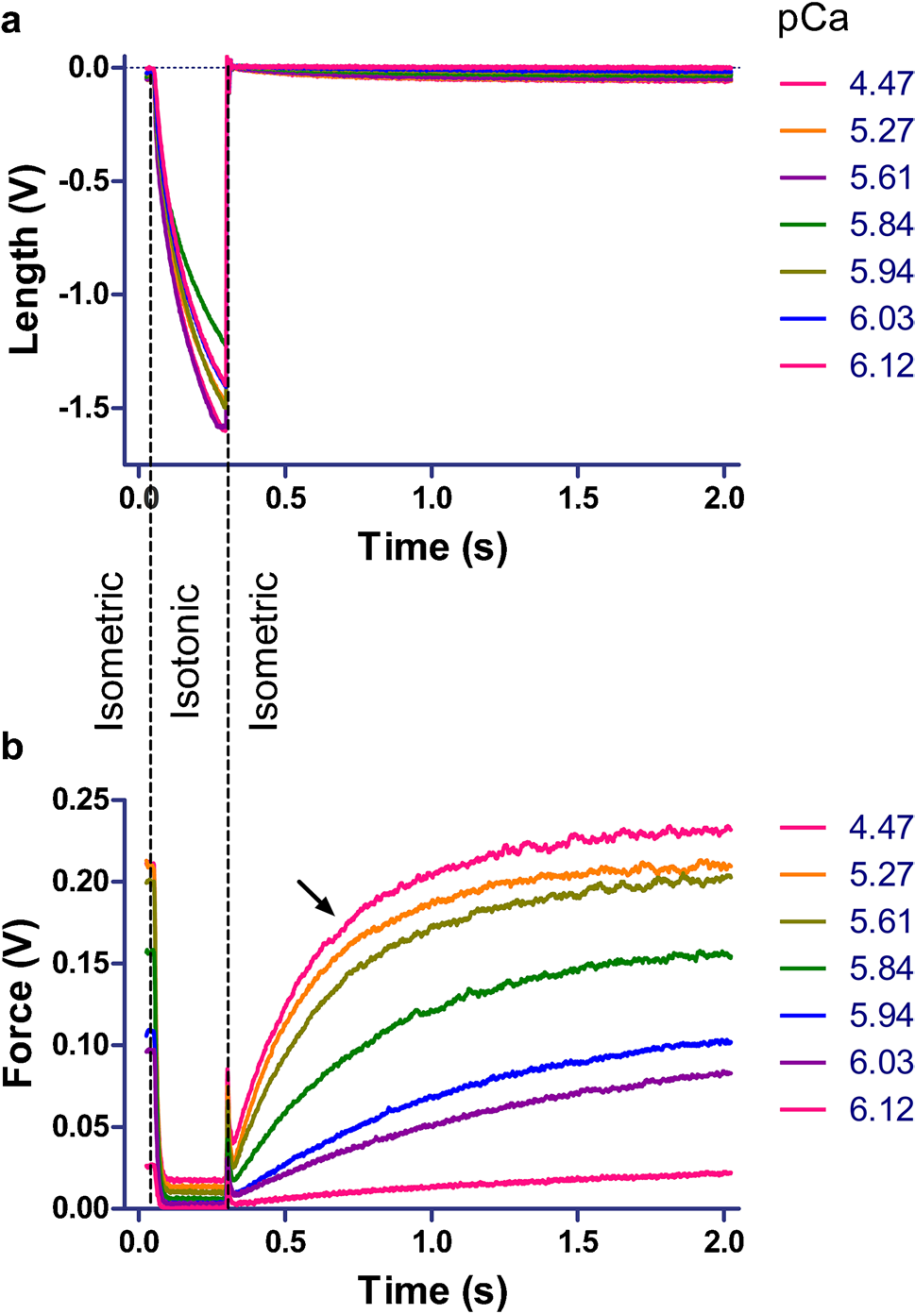
Skinned fiber length and force generation during isotonic and isometric contraction. **a** By modifying the position of the length driver, an isometric-held skinned fiber undergoes a transient phase of isotonic shortening (330 ms) and is then re-stretched to its original length, back to isometric contraction. The dotted lines show the time points of the switch from isometric to isotonic (slightly loaded) and back to isometric conditions. The procedure is performed in activating solution (pCa 4.47, i.e., saturating Ca^2+^ concentrations) and in different ratios of activating and relaxing solutions (i.e., sub-saturating Ca^2+^ concentrations). The described procedure is repeated several times for every pCa value and the resulting transients are added to generate representative or mean transients (essentially the same transients with even better signal-to-noise ratio; not shown). **b** Force transients corresponding to the length transients shown in A. Note the clear and fast reduction of force in the transition to the isotonic phase and the fast, pCa-dependent force redevelopment rate after switching back to isometric conditions. *k*_TRmax_ values were obtained by single-exponential fits of force transients during the force redevelopment phase observed in mean force transients at pCa 4.47 (arrow). For subsequent analysis (Fig. 2a, c, and e), isometric force at sub-saturating Ca^2+^ concentrations was normalized to isometric force obtained at pCa 4.47 in mean force transients

The above-described protocol was performed before and after incubating the mounted skinned fibers in 40 μg/mL Mt-I or Mt-II for 60 min in a relaxing solution. The apparatus used for these mechanical experiments is mounted on an inverted microscope, such that photos of specific sections of the isometric-held skinned fibers could be taken before and after toxin incubations. The experimental protocol is described in Fig. 1.

### Positive control for mechanical experiments using skinned fibers

The experiments described in the “Protocol for mechanical experiments” section and Fig. 1. were replicated by incubation of an additional set of skinned fibers in increasing concentrations of the myosin inhibitors AmBleb and BDM at pCa 4.47 (i.e., saturating Ca^2+^ concentration). Both myosin inhibitors influence the cross-bridge cycle of striated muscle, which in turn is reflected in several parameters of force development [19, 30, 35]. This set of experiments was designed as a positive control in order to show that the experimental protocol used here is sensitive to the effects of molecules that target and influence sarcomeric proteins.

### Experiments on myotubes

#### Differentiation of C2C12 myotubes

The cell culture was performed as described [1]. Briefly, C2C12 mouse progenitor myoblast cells (RRID:CVCL_0188) were seeded on 10 and 18 mm laminin-coated coverslips in DMEM/High Glucose (Capricorn Scientific DMEM-HA) with 1% penicillin and streptomycin (Capricorn Scientific PS-B) containing 15% fetal bovine serum (Gibco 10100147) until confluent (approx. 100,000 cells/cm^2^). Differentiation was induced by switching to a medium containing 2% horse serum (Gibco 26050088) for 6 days.

#### Myotoxin-induced morphological changes of myotubes

On the 6th differentiation day, plates containing myotubes were placed on an inverted microscope. The surface of the inverted microscope was previously set at 37°C. Thereafter, pre-warmed aliquots of culture medium containing Mt-I or Mt-II were gently added to the wells, achieving a final concentration of 40 μg/mL. Morphological changes of myotoxin-exposed myotubes were followed by bright-field microscopy for 60 min.

### Statistics and data analysis

All values are given as a mean ± SEM (standard error of the mean) with n representing the number of experiments. Mean values were compared using unpaired or paired Student’s *t*-test (*P *< 0.05) or one-way analysis of variance (ANOVA, *P*<0.05) and post hoc analyses of Tukey (multiple comparison test for single effects) as appropriate. Statistical analysis and figure layout were performed using GraphPad Prism (version 5.01; RRID:SCR_002798) for Windows, GraphPad Software, San Diego, CA, USA.

## Results

### Effect of Mt-I and Mt-II on single-skinned muscle fibers

**Fig. 2:**
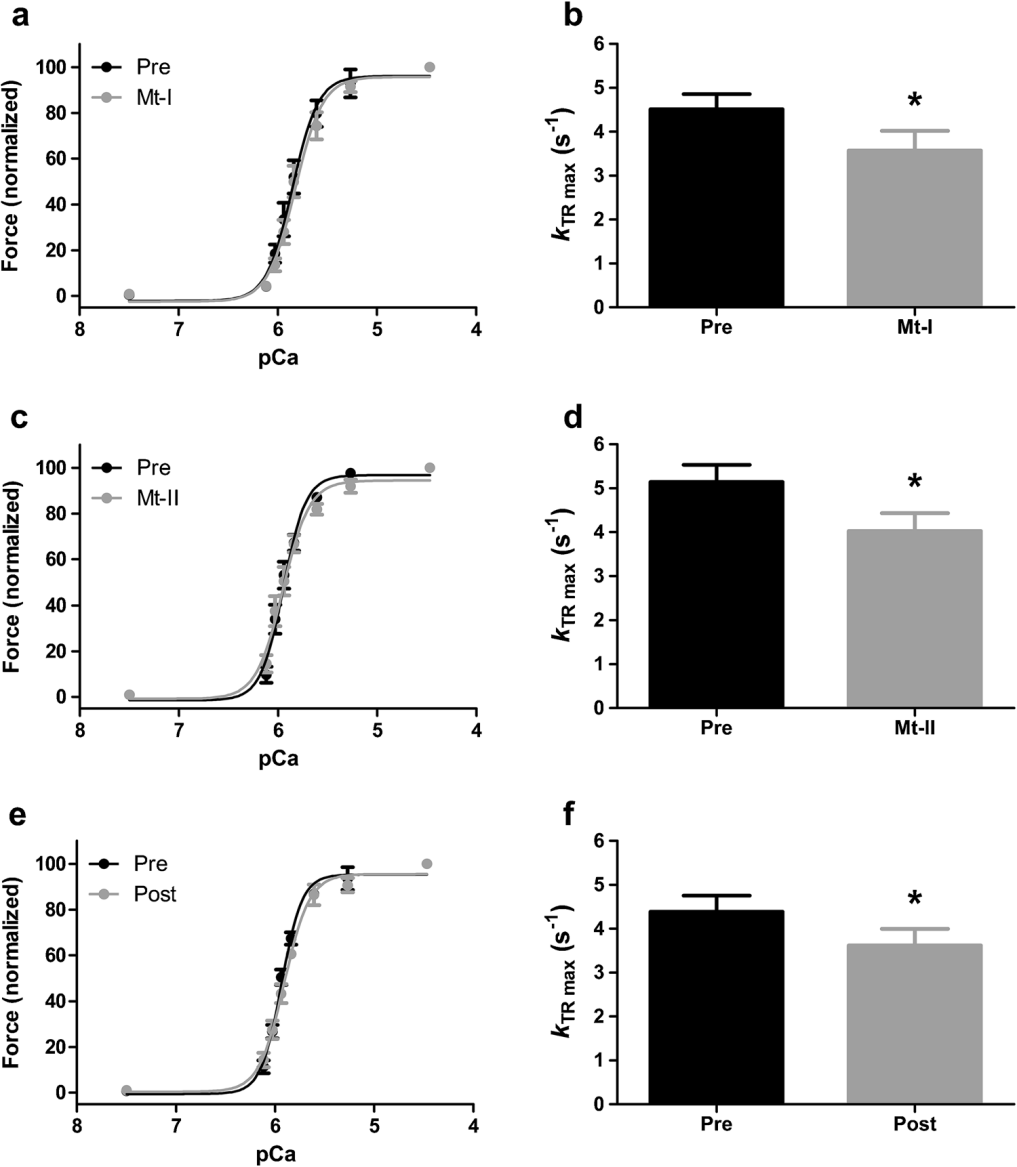
Effect of Mt-I and Mt-II on force-pCa relationship and *k*_TRmax_ of single-skinned muscle fibers. **a**,** c**, and **e** Force-pCa relationship of skinned fibers before and after being exposed to 40 μg/mL of either Mt-I (**a**) or Mt-II (**b**) for one hour in a relaxing solution. Ca^2+^-induced isometric force was not different before (Pre) and after (Mt-I, Mt-II) treatment. The same result was observed for control fibers undergoing the same procedure without Mt exposure (**e**). See Table 1. **b**,** d**, and **f** *k*_TRmax_: Rate constant of force development of skinned fibers at saturating Ca^2+^ concentration (pCa 4.47). *k*_TRmax_ was significantly lower after exposition to 40 μg/mL of either Mt-I (**b**) or MT-II (**d**) for one hour in a relaxing solution. However, a similar reduction was observed on control fibers undergoing the same procedure without Mt exposure (**f**). *: significantly lower *k*_TRmax_ than in the corresponding Pre condition. See bold entries in Table 1

**Fig. 3:**
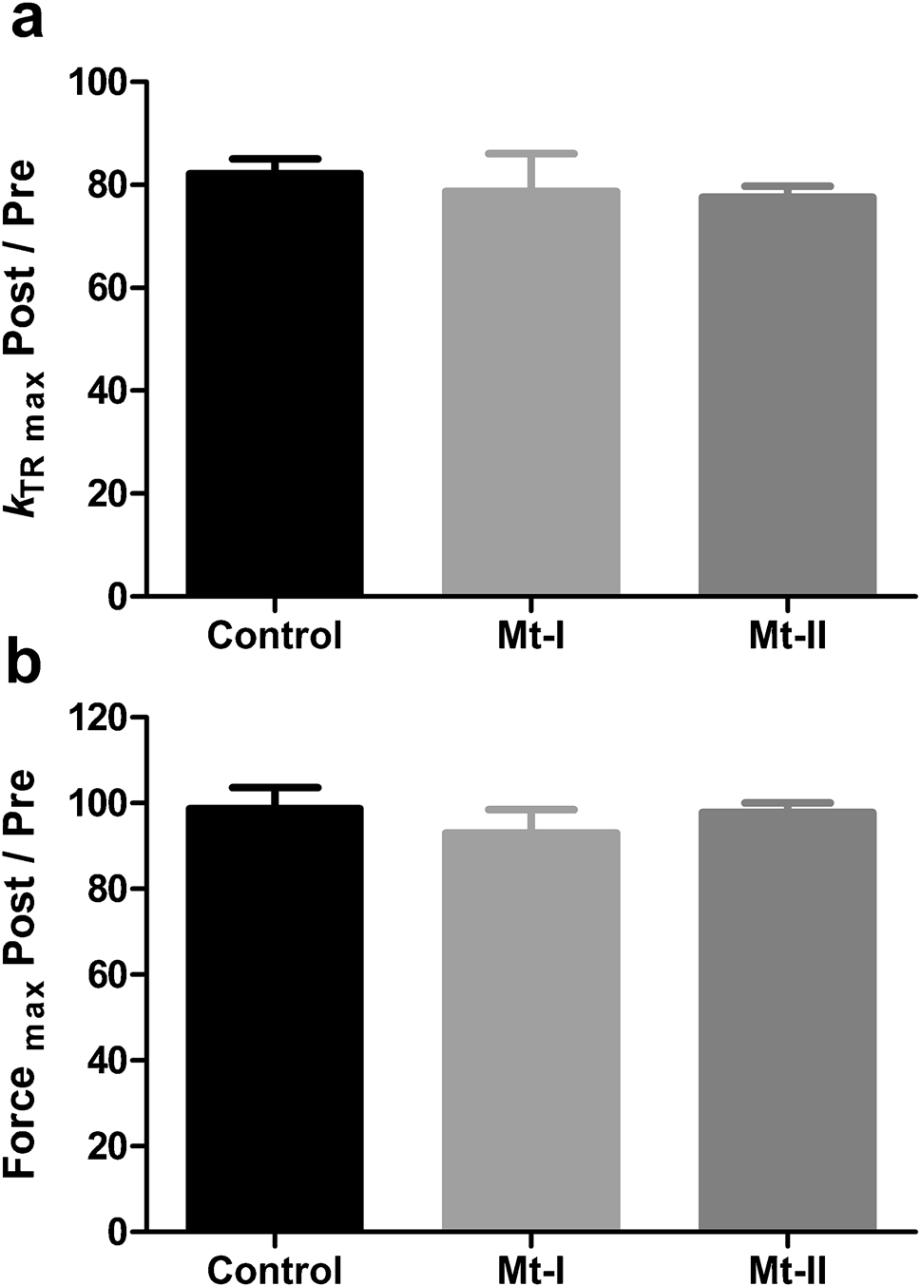
Relative changes of *k*_TRmax_ and Force_max_ after incubation with myotoxins. **a** *k*_TRmax_ of skinned fibers after being exposed to 40 μg/mL myotoxins or control incubation for one hour in relaxing solution as normalized to its corresponding magnitude before incubation. **b** Isometric force at saturating Ca^2+^ concentration (pCa 4.47) of skinned fibers after being exposed to 40 μg/mL myotoxins or control incubation for one hour in relaxing solution as normalized to its corresponding magnitude before incubation. See Table 2

**Fig. 4:**
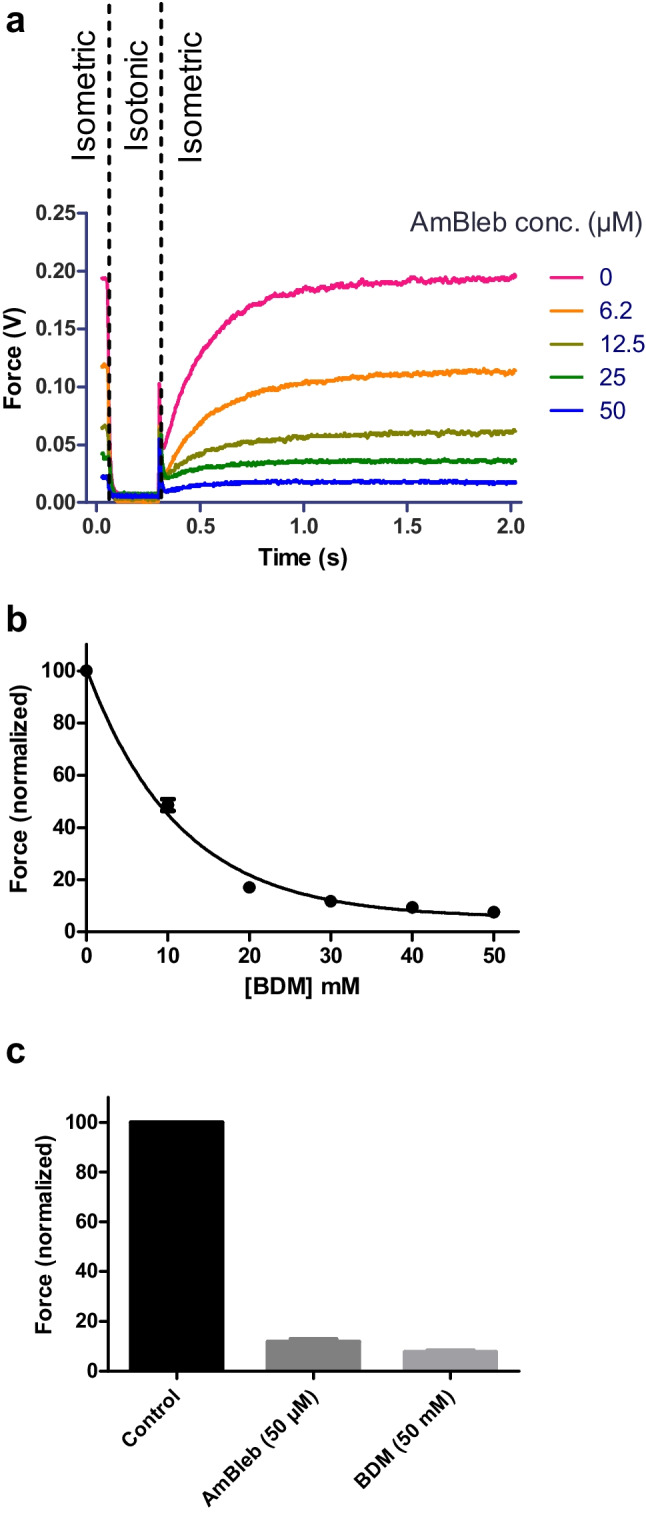
Effect of myosin inhibitors on force development. **a** Original force redevelopment transients recorded at saturating Ca^2+^ concentration. Experimental conditions correspond to the top transient shown in Fig. 1b (pCa 4.47). Note how increasing concentrations of the myosin inhibitor AmBleb (subsequent transients at 6.2, 12.5, 25, and 50 μM [AmBleb]) reduce total force generation, even though the Ca^2+^ concentration remains the same. **b** Same experimental protocol as in a., but using a different myosin inhibitor (BDM). Instead of the original force transients, only the resulting force development as a function of [BDM] is shown. As in the case of AmBleb, BDM clearly reduces force generation at saturating Ca^2+^ concentration in a dose-dependent manner (*n* = 7). **c** Comparison of maximum force inhibition achieved by AmBleb and BDM. At those concentrations, force inhibition becomes asymptotic (as shown in b.), such that further increases in the myosin inhibitors did not induce additional force inhibition (*n* = 5 for AmBleb and 7 for BDM)

**Fig. 5:**
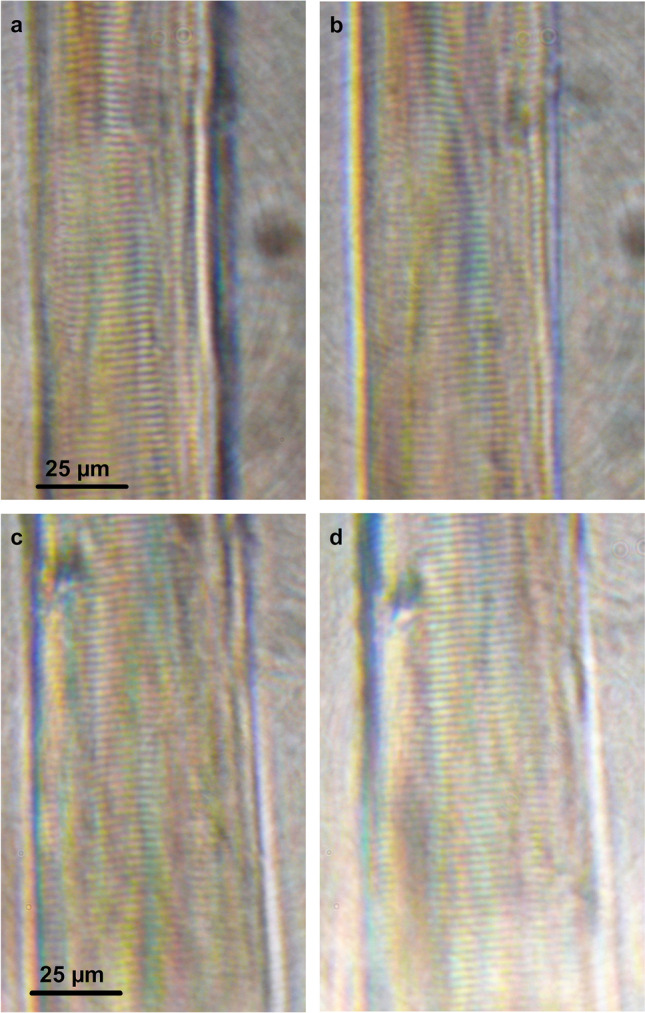
Effect of Mt-I and Mt-II on sarcomeric morphology of isometric-held, single-skinned muscle fibers. **a, b** No changes in sarcomeric morphology were observed by light microscopy before (**a**) and after (**b**) incubation with 40 μg/mL Mt-I for one hour in a relaxing solution. **c, d** Same as in a, b for Mt-II

To evaluate the effects of myotoxins on the contractile apparatus of striated muscle, single-skinned muscle fibers were incubated with 40 μg/mL Mt-I or Mt-II for 60 min in a relaxing solution. The results of these experiments are summarized in Fig. 2 and Table 1. Figure 2a and c show the normalized force development of single-skinned fibers before and after incubation with Mt-I or Mt-II, respectively. The exposure to myotoxins did not influence Ca^2+^ sensitivity nor cooperativity of force development, as evidenced by the similar pCa_50_ values and Hill coefficients (*n*_H_) before and after myotoxin treatment in both experiments (Table 1). On the other hand, the rate constant of force redevelopment at saturating Ca^2+^ concentration (*k*_TRmax_) was significantly lower after Mt-I or Mt-II incubation, as shown in Figs. 2b and d, respectively. To control whether *k*_TRmax_ decrease was induced by myotoxins or alternatively by the experimental treatment itself (i.e., two full sets of Ca^2+^-induced activations, including numerous shortening – re-stretch cycles), the experiment was replicated by incubating a third group of skinned fibers in relaxing solution for 60 min without toxins. This experiment also controls possible masked effects in the experiments presented in Figs. 2a and c (i.e., opposite, cancelling effects of the experimental treatment and toxins on pCa_50_ values and Hill coefficients). As shown in Fig. 2e and Table 1, the results of this additional control experiment are essentially the same as in the experiments including myotoxin treatment (Figs. 2a and c), confirming the absence of any myotoxin-induced effect on Ca^2+^-sensitivity or cooperativity of force development. Additionally, Fig. 2f shows a significant decrease of *k*_TRmax_ in this toxin-free control experiment, confirming that this change is not induced by the toxins, but by the experimental protocol. To control for a possible additive effect of toxins and treatment on *k*_TRmax_ results, the relative magnitude of *k*_TRmax_ decrease (i.e., ratio Post-/Pre-incubation; *k*_TRmax_ Post/Pre) in all three experiments was calculated (Fig. 3a). For both toxin and toxin-free experiments, the observed *k*_TRmax_ decrease was always ~20 % and non-significant differences were detected (Table 2). Finally, Fig. 3b shows maximal force development at saturating Ca^2+^ concentration (pCa 4.47) after myotoxin or myotoxin-free incubation as compared to its magnitude before incubation (i.e., ratio Post-/Pre-incubation; Force_max_ Post/Pre). As for all the previously analyzed variables, no statistically significant differences were detected. In contrast to the lack of effect of both myotoxins on skinned fibers, the set of experiments summarized in Fig. 4 using myosin inhibitors shows that the experimental protocol is able to detect the effects of agents that affect sarcomere proteins. This positive control rules out the possibility of a lack of detection capability in the experiments performed. Altogether, this set of mechanical experiments strongly suggests that Mt-I and Mt-II do not affect the main parameters of force development in striated muscle sarcomeres. In line with the mechanical experiments, bright field microscopy of segments of the skinned fibers used for experiments shown in Figs. 2a and c does not evidence structural changes after incubation with myotoxins (Fig. 5).

**Table 1:** Effect of Mt-I and Mt-II on force parameters of single skinned muscle fibers: absolute values

	Mt-I, 40 μg/mL			Mt-II, 40 μg/mL			Control		
	Pre	After Mt-I incubation	*P* values	Pre	After Mt-II incubation	*P* values	Pre	After control incubation	*P* values
pCa_50_	5.84 ± 0.04	5.81 ± 0.04	0.1397	5.95 ± 0.02	5.95 ± 0.03	0.6701	5.94 ± 0.01	5.88 ± 0.04	0.2320
*n*_H_	3.67 ± 0.60	3.81 ± 0.64	0.3080	3.98 ± 0.40	3.45 ± 0.21	0.1295	3.83 ± 0.25	3.10 ± 0.19	0.1528
*k*_TRmax_	4.51 ± 0.34	3.57 ± 0.44	**0.0333**	5.14 ± 0.39	4.02 ± 0.40	**0.0001**	4.39 ± 0.36	3.61 ± 0.38	**0.0093**

pCa_50_: -LOG_10_ of Ca^2+^ concentration at 50% of maximal force generation. *n*_H_: Hill coefficient; slope of the force−pCa relationships presented in Figs. 2a, c, and e. *k*_TRmax_: Rate constant of force development at saturating Ca^2+^ concentration (pCa 4.47). Data represent mean ± SEM. n = 5 in all conditions. Bold entries: statistic significant (*P *< 0.05)

**Table 2:** Effect of Mt-I and Mt-II on *k*_TRmax_ and Force_max_ of single-skinned muscle fibers: relative changes

	Control incubation	Mt-I, 40 μg/mL	Mt-II, 40 μg/mL	F ratio and *P* values
*k*_TRmax_ Post/Pre	82.1 ± 2.94	78.7 ± 7.31	77.6 ± 2.08	0.20 / 0.817
Force_max_ Post/Pre	98.8 ± 4.90	93.1 ± 5.42	97.7 ± 2.37	0.48 / 0.629

*k*_TRmax_ Post/Pre: Rate constant of force development at saturating Ca^2+^ concentration (pCa 4.47) after being exposed to 40 μg/mL myotoxins or control incubation for one hour in relaxing solution as normalized to its corresponding magnitude before incubation. Force_max_ Post/Pre: Isometric force at saturating Ca^2+^ concentration (pCa 4.47) of skinned fibers after being exposed to 40 μg/mL myotoxins or control incubation for one hour in relaxing solution as normalized to its corresponding magnitude before incubation. Data represent mean ± SEM. *n* = 5 in all conditions

### Effect of Mt-I and Mt-II on myotubes

**Fig. 6:**
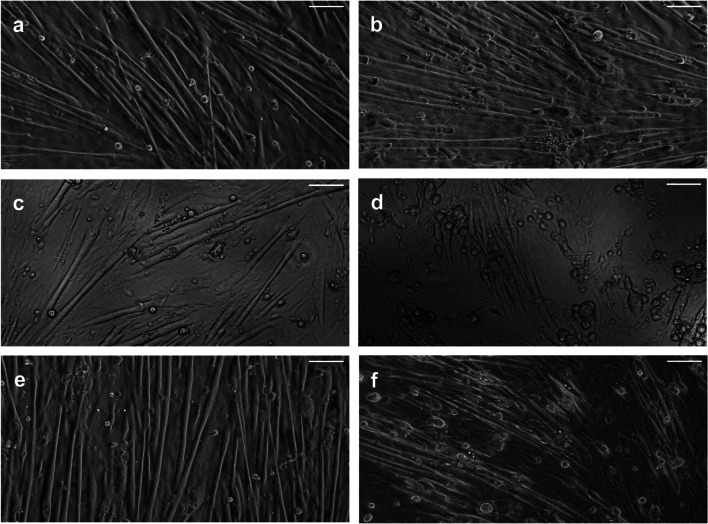
Effect of Mt-I and Mt-II on morphology of C2C12 myoblast-derived myotubes. **a**, **b** No changes in myotubes morphology were observed by light microscopy before (**a**) and after (**b**) 60 min control incubation. **c**, **d** Clear differences in myotubes morphology were observed before (**c**) and after (**d**) incubation with 40 μg/mL Mt-I for 60 min. **e**, **f** Same as in c, d for Mt-II. Myotoxins induced a strong hypercontraction of myotubes. White bar 100 μm. See online resources 1 and 2

In order to contrast the lack of effects caused by myotoxins on skinned muscle fibers compared to intact skeletal muscle cells with cell membranes, myotubes differentiated from mouse C2C12 myoblasts progenitor cells were exposed to either Mt-I or Mt-II. These experiments are summarized in Fig. 6. In this cell model, both myotoxins induced a strong hypercontraction leading to clear morphological alterations. Hypercontraction originated at a particular point of the cell and was followed by a retraction along its longitudinal axis. Online resources 1 and 2 show an example of the typical hypercontraction of myotubes after myotoxin exposure.

## Discussion

In spite of significant advances towards characterizing and understanding the effects and mode of action of snake venom sPLA_2_ toxins [15, 25], their molecular, functional, and structural details have been only partially elucidated. In the case of myotoxic sPLA_2_s from viperids, compelling evidence supports a membrane-permeabilizing action leading to a massive increase of cytoplasmic Ca^2+^, both for enzymatically active (Asp49) sPLA_2_s and enzymatically inactive (Lys49) sPLA_2_-like homologs [7, 8, 32, 40]. Consequent to this Ca^2+^ increment, a rapid and strong hypercontraction is observed in skeletal muscle fibers after local injection of these myotoxins in mice [17, 26]. Interestingly, recent studies have revealed that the myotoxins are rapidly internalized in myogenic cells *in vitro* [31] and in mature skeletal muscle fibers *in vivo* [38], underscoring the relevance of assessing their possible effects on intracellular targets, in addition to their membranotropic actions.

Skinned muscle cells represent a useful *ex vivo* model to discern between direct and indirect actions of biomolecules on the contractile apparatus and have been recently used to compare the effects of a Lys49 sPLA_2_ homolog, Mt-II, on isolated intact vs. skinned rat cardiomyocytes [29]. Exposure to Mt-II led to a rapid cytosolic Ca^2+^ increase and hypercontraction in intact cardiomyocytes, but did not have effects on the contractile apparatus of the skinned cells. Aiming to extend these observations to skeletal muscle and to a catalytically active myotoxin, the present study investigated the effects of Mt-I and Mt-II on rabbit skinned skeletal muscle fibers. Exposure of skinned fibers to these myotoxins did not induce any direct alterations of parameters such as Ca^2+^ sensitivity or cooperativity of force development. A slight decrease recorded for the rate constant of force redevelopment (*k*_TRmax_) after incubation with the myotoxins is attributable to the experimental treatment *per se*, since it was also observed in control experiments in the absence of toxins.

Experiments with myosin inhibitors showed a strong force inhibition on skinned fibers. AmBleb is a derivative of blebbistatin that prevents myosin from entering the force-generating states by stabilizing the closed state of the switch 2 element of the nucleotide-binding site. In this state, both ADP and Pi are bound to the active site and the formation of the strong actomyosin interaction is inhibited [35, 39, 42, 44]. BDM is also a widely used myosin inhibitor. Recent research has suggested that BDM accelerates ATP cleavage of skeletal myosin subfragment 1 and alters the microenvironment around the phosphorus atoms of myosin-bound ATP analogs, thereby inhibiting active force generation [19]. Our positive controls with both inhibitors confirm the suitability of our setup and experimental protocol to detect changes at the level of the cross-bridge cycle. In addition, we have previously shown that myosin inhibitors can also modify other key variables of force development in our skinned fiber preparation, including its Ca^2+^ sensitivity and cooperativity (i.e., pCa_50_ and *n*_H_ values, respectively) [30].

By microscopical assessment, no morphological changes were evidenced in segments of skinned fibers exposed to the myotoxins. Therefore, it can be concluded that neither Mt-I nor Mt-II has direct effects on the contractile machinery of skeletal muscle fibers and that hypercontraction of fibers results from indirect effects, i.e., the large cytosolic Ca^2+^ increase after sarcolemma permeabilization. Such hypercontraction observed in skeletal muscle tissue [17, 26] was readily reproduced in myotubes differentiated *in vitro* from mouse C2C12 myoblasts, after exposure to either Mt-I or Mt-II. It remains to be determined whether these myotoxins, once internalized, affect other intracellular structures, such as the sarcoplasmic reticulum and mitochondria, and whether these alterations contribute to cellular damage. Moreover, the mechanical disruptive effect of hypercontraction in the integrity of the plasma membrane is another possible effect that might contribute to cell damage and needs to be investigated.

## Supplementary information

ESM 1 Online Resource 1 Baseline: Myotubes morphology observed by light microscopy 1 min after incubation with 40 μg/mL Mt-II. The myotoxin has not exerted its effect.

ESM 2 Online Resource 2 Clear differences in some myotubes morphology were observed after 17 min incubation with 40 μg/mL Mt-II. Note the fast hypercontraction of some myotubes.

## Data Availability

The datasets generated during the present study are available from the corresponding author upon reasonable request.
